# Expansion of NK Cells and Reduction of NKG2D Expression in Chronic Lymphocytic Leukemia. Correlation with Progressive Disease

**DOI:** 10.1371/journal.pone.0108326

**Published:** 2014-10-06

**Authors:** Leticia Huergo-Zapico, Andrea Acebes-Huerta, Ana Pilar Gonzalez-Rodriguez, Juan Contesti, Esther Gonzalez-García, Angel R. Payer, Monica Villa-Alvarez, Azahara Fernández-Guizán, Alejandro López-Soto, Segundo Gonzalez

**Affiliations:** 1 Department of Functional Biology, Universidad de Oviedo, Oviedo, Spain; 2 IUOPA, Universidad de Oviedo, Oviedo, Spain; 3 Department of Hematology, Hospital Universitario Central de Asturias, Oviedo, Spain; 4 Department of Hematology, Hospital Cabueñes, Gijón, Spain; University of Manitoba, Canada

## Abstract

The immune system may mediate anti-tumor responses in chronic lymphocytic leukemia (CLL) which may affect disease progression and survival. In this study, we analyzed the immune characteristics of 99 consecutive previously diagnosed CLL patients and 50 healthy controls. The distribution of lymphocyte subsets at diagnosis was retrospectively analyzed. Compared with controls, leukemia patients showed an expansion of NK and CD8 T cells at diagnosis. The relative number of CD8 T cells at diagnosis was associated with time to treatment, suggesting that CD8 T cells may modify disease progression. The distribution of lymphocyte subsets was analyzed again when patients were enrolled in this study. The median time since these patients were diagnosed was 277 weeks. Compared with diagnosis, the absolute number of CD8 T cells significantly decreased in these patients, reaching similar values to healthy controls; however NK cells kept significantly elevated overtime. Nevertheless, NK cells showed an impaired expression of NKG2D receptor and a defective cytotoxic activity. This down-regulation of NKG2D expression was further enhanced in patients with advanced and progressive disease. Additionally, membrane NKG2D levels significantly decreased on CD8 T cells, but a significant increase of NKG2D+CD4+ T cells was observed in CLL patients. The cytotoxic activity of NK cells was diminished in CLL patients; however the treatments with IL-2, IL-15, IL-21 and lenalidomide were able to restore their activity. The effect of IL-2 and IL-15 was associated with the increase of NKG2D expression on immune cells, but the effect of IL-21 and lenalidomide was not due to NKG2D up-regulation. The expansion of NK cells and the reversibility of NK cell defects provide new opportunities for the immunotherapeutic intervention in CLL.

## Introduction

Chronic lymphocytic leukemia (CLL) is the most common adult leukemia in Western countries. It is characterized by a clonal accumulation of mature malignant B cells in blood, bone marrow and lymphoid organs. There is a marked clinical heterogeneity in this disease that is associated with a heterogeneous array of genetic and molecular defects [1]. The complexity of this malignancy is further increased by the interaction of leukaemia cells with the microenvironment [2]. Leukaemia cells closely interact with accessory and immune cells that regulate their trafficking, survival and proliferation [3]. Additionally, the immune system may mediate anti-tumor responses in CLL which may affect disease progression and survival [4]–[6]. Nevertheless, patients progressively develop multiple immune defects, including hypogammaglobulinemia, impairment of the function of T, NK and dendritic cells, as well as alterations in the cytokine network [7]. Likewise, patients with advanced disease frequently develop a severe immunodeficiency.

NKG2D is an activating receptor expressed by NK and T cells that plays a key role in the immune response against cancer [8], [9]. NKG2D is the receptor for MHC class I-related chain A and B (MICA/B) and UL16-binding proteins 1–6 (ULBP1-6), which are restrictedly expressed in benign cells, but are up-regulated in stressed and transformed cells, triggering a potent anti-tumour immune response [10]–[12]. Leukaemia cells of CLL patients express low membrane levels of NKG2D ligands and shed soluble NKG2D ligands, which confers poor prognosis to CLL patients [13], [14]. Accordingly, a reduction of NKG2D expression on CD8 T cells in a cohort of CLL patients with high levels of serum soluble MICA (sMICA) has been reported [15].

In this study, we analyzed the evolution of the number and the characteristics of the immune cells with the progression of CLL. We also examined the expression of NKG2D receptor on these cells, which may play a key role in the anti-tumor activity against leukemia cells.

## Material and Methods

### Patient and CLL samples

99 consecutive previously diagnosed CLL patients and 50 healthy matched controls were analyzed in this study (Table 1). Patients were diagnosed between 1982 and 2011. The median time since they were diagnosed was 277 weeks. As previously described, patients were classified as having stable (n = 38) or progressive disease (n = 61) [16]. 27 patients had received chemotherapeutic treatment; however none of them received any treatment 6 months before being enrolled in this study.

**Table 1:** Clinical characteristics of CLL patients.

Characteristic	n = 99
Age at diagnosis (years)	68,2
Gender: Male/Female	63/36
Rai stage at diagnosis (%)	
Low: 0/I	45
Intermediate: II/III	33
High IV/V	21
Binet	
A	67
B	15
C	17
Progressive/stable disease	61/38
Lymphocytes (x10^9^/L)	13.2 (0,6–300.1)*
Affected Lymph nodes	
0	58
1	15
2	14
≥3	12
ECOG	
0–1	69
2	22
3	8
4	2
CD38 (%)**	20%
Gammaglobulins (gr/L)	9.0 (4–20.1)*
IgG (gr/L)	9.39 (3.6–21.7)*
IgA (gr/L)	1.6 (0.1–4.4)*
IgM (gr/L)	0.5 (0.1–4)*
LDH (U/L)	287 (142–928)*
β2-microglobulin (mg/L)	3.14 (0.9–18)*
MBC duplication in less than 1 year (%)	32%

MBC: monoclonal B-cells clone.

* median and range.

** Positive (>30%).

Immunological characteristics of these patients at diagnosis were retrospectively analyzed. Clinical and immunological characteristics of the patients were analyzed when patients were enrolled in this study. For that purpose, peripheral blood mononuclear cells (PBMCs) from freshly isolated blood obtained from patients and controls were purified by Ficoll-Paque (Pharmacia) density centrifugation. NK cells were isolated using the EasySep NK Cell Enrichment kit (StemCell Technologies). This study was approved by the Hospital Universitario Central de Asturias Ethics Committee and written informed consent was obtained from all patients and controls.

### Flow cytometry

Diagnosis of CLL for each patients was confirmed by flow cytometry, which revealed a typical CD19+, CD20+, CD5+, CD23+ and Ig light chain (κ or λ) restricted phenotype. Absolute counts of the main PBMCs subsets, including CD4 and CD8 T cells, B cells and NK cells were carried out by flow cytometry upon diagnosis. The distribution of these subsets of lymphocytic cells and the membrane expression of NKG2D were also analyzed at the time of patient enrollment. The protocol for B-cell chronic lymphoproliferative diseases used in all cases included the following antibody conjugates: anti-CD3-FITC, anti-CD4-PerCP, anti-CD8-CF-Blue, anti-CD56-APC, anti-CD19-APC (all from Immunostep), anti-CD3-PECy7 (eBioscience) and anti-NKG2D-PE (Miltenyi Biotec). The populations of cells were defined as follows: CD4 T cells were defined as CD3+CD4+, CD8 T cells were defined as CD3+CD8+, B cells were defined as CD19+ and NK cells were defined as CD3-CD56+. Cells were analyzed on a BD Biosciences FACSCanto II cytometer and data were analyzed by FACSDiva software (Beckton Dickinson).

### Cell lines and cell cultures

K562 cells were obtained from ATCC and were grown in RPMI-1640, 2 mM L-glutamine and gentamicin (complete media) supplemented with 10% heat-inactivated human AB serum. 2×10^6^ PBMCs were cultured in complete media containing 10% of human AB serum in the presence of IL-2 (5 ng/ml), IL-15 (25 ng/ml), IL-21 (20 ng/ml) (all from Peprotech) for 48 h or lenalidomide (1 µM) (Celgene) for 7 days. Cultured cells were incubated with a mixture of fluorescent-labelled antibodies: anti-CD3-FITC, anti-CD4-PerCP, anti-CD8-CF-Blue, anti-CD56-APC or anti-NKG2D-PE for 15 min. After washing, cells were analyzed by flow cytometry.

### CD107a degranulation assay and cytotoxic assay

CD107a lysosome-associated membrane protein-1 (LAMP-1) was used to measure NK, NKT-like and CD8 cell cytotoxic activity. PBMCs from healthy donors stimulated with IL-2 (5 ng/ml), IL-15 (25 ng/ml), IL-21 (20 ng/ml) for 48 hours or lenalidomide (1 µM) for 7 days were incubated with target K562 cells at an Effector: Target (E:T) ratio of 5∶1 in complete media supplemented with human AB serum and BD GolgiStop (BD Biosciences). As a positive control of degranulation, PBMCs were stimulated with PMA (50 ng/ml) and ionomycin (250 ng/ml) (both from Sigma-Aldrich). The anti CD107a-PE antibody (BD Biosciences) was added to the plate during the incubation. After the incubation, samples were stained for CD3, CD4, CD8 and CD56 expression and were analyzed by flow cytometry.

### Statistical analysis

Gaussian distribution of values was tested by Kolmogorov-Smirnov test. Relationship between continuous variables and categorical prognostic variables was evaluated by Mann–Whitney and Kruskal–Wallis tests. The Wilcoxon Matched-Pairs Signed Ranks test was utilized for intra-group comparisons. Spearman's rank correlation test was used to analyze the correlation between the biological parameters. Time to treatment was defined as the time from diagnosis to time of first therapy. Time to treatment was analyzed using the approach of Kaplan and Meier. P-values <0.05 were considered significant. All analyses were conducted with SPSS, version 15.0.

## Results

### CD8 T cells and NK cells populations were expanded at diagnosis of CLL

**Figure 1:**
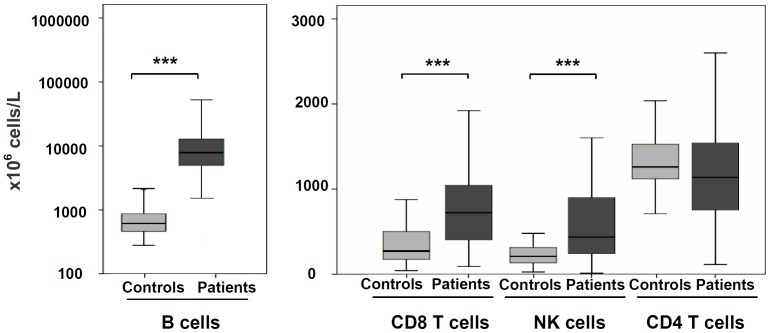
Absolute numbers of immune cells at diagnosis of CLL. Absolute numbers of B cells (CD19+), CD4 T cells (CD3+CD4+), CD8 T cells (CD3+CD8+) and NK cells (CD3-CD56+) at diagnosis of 99 CLL patients were compared with 50 healthy controls. Horizontal bars, boxes and whiskers represent median, 25%/75% quartiles and range, respectively. (***p<0.001).

99 consecutive previously diagnosed CLL patients and 50 healthy matched controls were analyzed in this study (Table 1). The distribution of lymphocyte subsets in these patients at diagnosis was retrospectively analyzed. At diagnosis, additionally to an increase of B cells, CLL patients showed a marked increase of NK cells (median of 0.436 vs. 0.209×10^9^ cells/L; p<0.0001) and T cells (2.048 vs. 1.67×10^9^ cells/L; p<0.0001) compared with controls (Fig. 1). There was an increase of CD8 T cells (0.723 vs. 0.271×10^9^/L; p<0.0001), but no differences were observed in CD4 T cells. Of note, significant correlations between the absolute numbers of B cells at diagnosis and the numbers of T cells (r = 0.31, p = 0.02), CD4 T cells (r = 0.274, p = 0.01), CD8 T cells (r = 0.362, p = 0.01) and NK cells (r = 0.246, p = 0.02) were observed.

### CD8 T cells, but not NK cells, decreased with the progression of CLL

**Figure 2:**
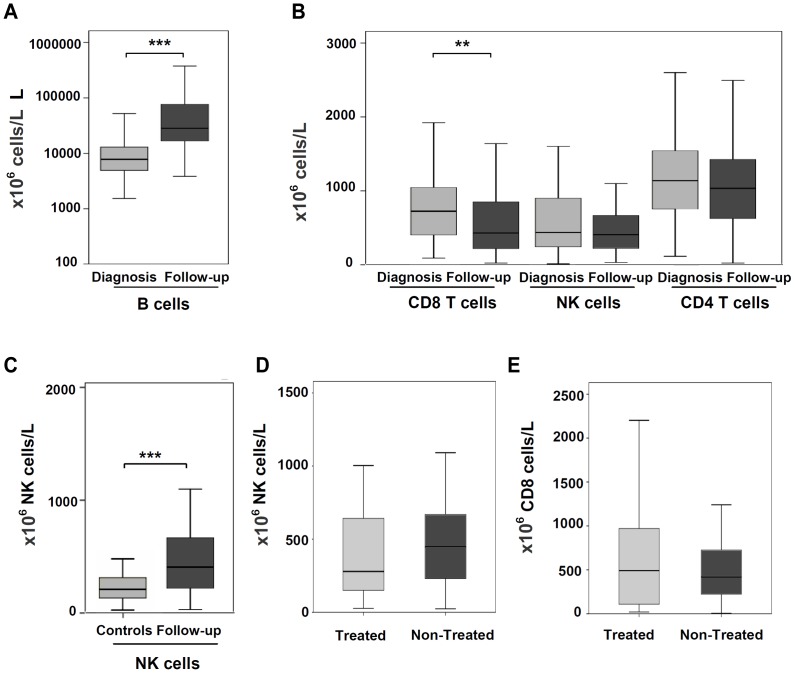
Effect of the disease progression on the absolute numbers of B cells, CD4 T cells, CD8 T cells and NK cells. (A and B) Absolute numbers of B cells, CD8 T cells, NK cells and CD4 T cells were re-evaluated after the evolution of the disease, and they were compared with the values at diagnosis. The median time since the patients were diagnosed was 277 weeks. (C) Absolute NK cell number at the follow-up evaluation was compared with healthy controls. (D and E) Comparison of the absolute numbers of CD8 T cells and NK cells at the follow-up evaluation, according to the chemotherapy treatment. (**p<0.01; ***p<0.001).

The distribution of lymphocyte subsets in patients was again analyzed by flow cytometry when patients were enrolled in this study. The median time since the patients were diagnosed was 277 weeks. At that stage of the disease, patients showed a significant increase of B cells compared with the absolute numbers at diagnosis (Fig. 2A). A concomitant decrease in the absolute number of CD8 T cells (0.419 vs. 0.723×10^9^/L, p<0.0001), reaching values similar to healthy controls (0.419 vs. 0.271×10^9^/L), was observed (Fig. 2B). No differences in CD4 T cells were detected. Similar to diagnosis, the number of NK cells kept significantly higher than controls (0.398 vs. 0.209×10^9^/L; p<0.0001) (Fig. 2B and 2C).

The changes in the lymphocyte subsets overtime were also analyzed in relation with the clinical characteristics of the patients. The decrease of CD8 T cells was not significantly different between patients with progressive and stable disease (not shown). There were also no differences in relation with the chemotherapy treatment. Patients who were previously treated (n = 27) showed a non-significant decrease of NK cells (0.636 vs. 0.377×10^9^/L) and, a slight increase in CD8 T cells (0.566 vs. 0.643×10^9^/L) compared with non-treated ones, (Fig. 2D and E).

26 CLL patients developed a second malignancy, mainly skin (n = 8), prostate (n = 4), colon (n = 3) and bladder (n = 3) cancers. Compared with the absolute numbers at diagnosis, those patients who developed a second malignancy had a higher loss in the absolute numbers of NK cells (−0.139 vs. −0.122×10^9^/L, p = 0.04), CD8 T cells (−0.668 vs. −0.293×10^9^/L; p = 0.1) and CD4 T cells (−0.197 vs. −0.077×10^9^/L; p = 0.4) than those who did not suffer a second malignancy, in that deterioration of the immune system may be associated with the development of these tumors.

### Prognosis significance of CD8 count in CLL patients

**Figure 3:**
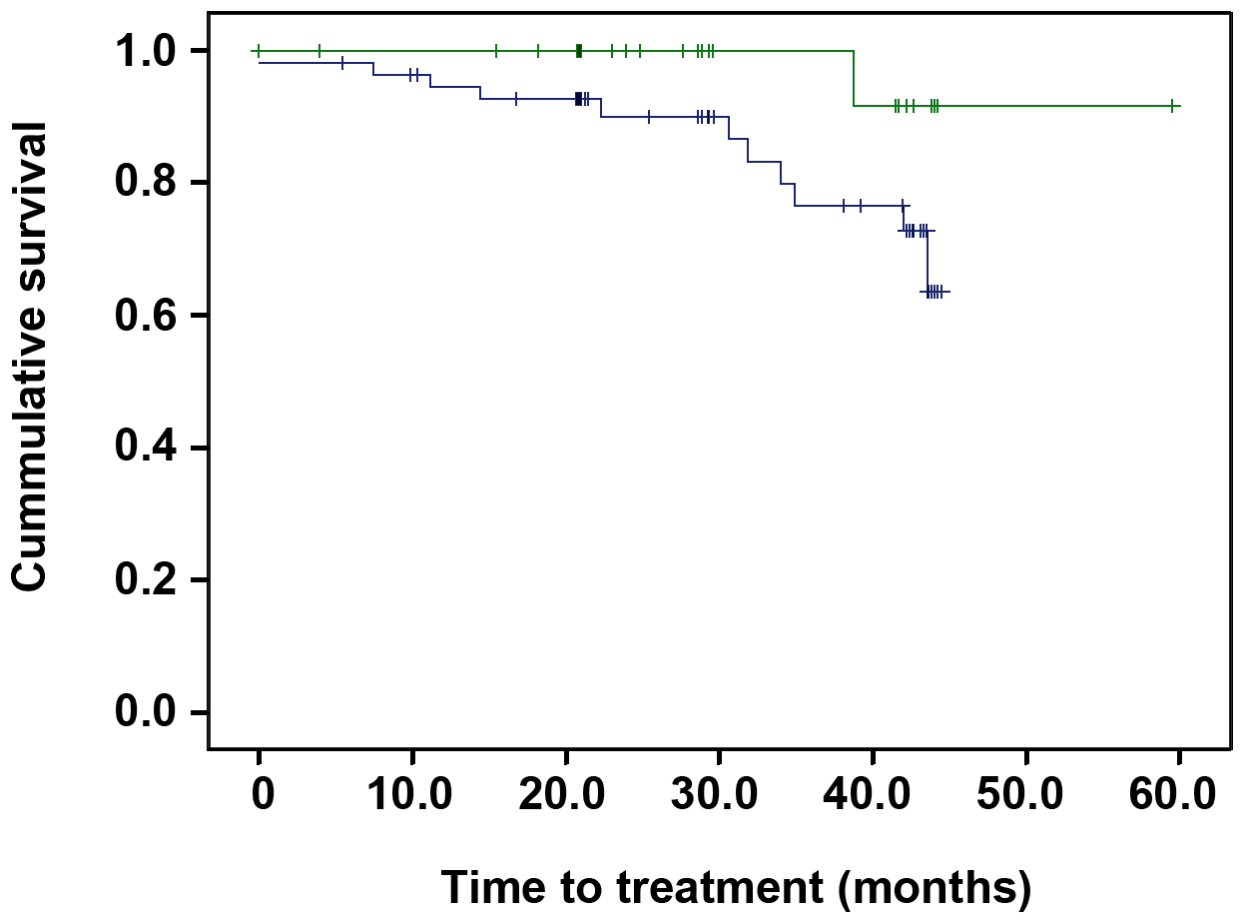
Prognostic significance of CD8 T cells in CLL. Kaplan-Meier curves were used to analyze the prognostic significance of the relative numbers of CD8. The relative number of CD8 T cells were analyzed by calculating the ratio between the numbers of CD8 T cells and Monoclonal B Clone (MBC) (T/NK cells: MBC ratio) as previously described (4). Patients with relative CD8 T cell count >0.03 (green line) showed significantly higher time to treatment than those with lower numbers of CD8 T cells (blue line). Statistical differences were analyzed by long-rank test.

The relative numbers of T and NK cells at diagnosis have been associated with clinical outcome in CLL [4], [5]. Thus, we analyzed whether the relative numbers of T and NK cells at diagnosis influence the time to treatment. The relative numbers of T cells and NK cells were analyzed by calculating the ratio between the numbers of T/NK cells and Monoclonal B Clone (MBC) (T/NK cells: MBC ratio) as previously described [4]. Patients with relative CD8 T cell count >0.03 showed a significantly higher time to treatment than those with lower numbers of CD8 T cells (Fig. 3), suggesting that CD8 T cells may modify disease progression. No other association was observed.

### NKG2D membrane expression is decreased on NK cells and increased on CD4 T cells of CLL patients

**Figure 4:**
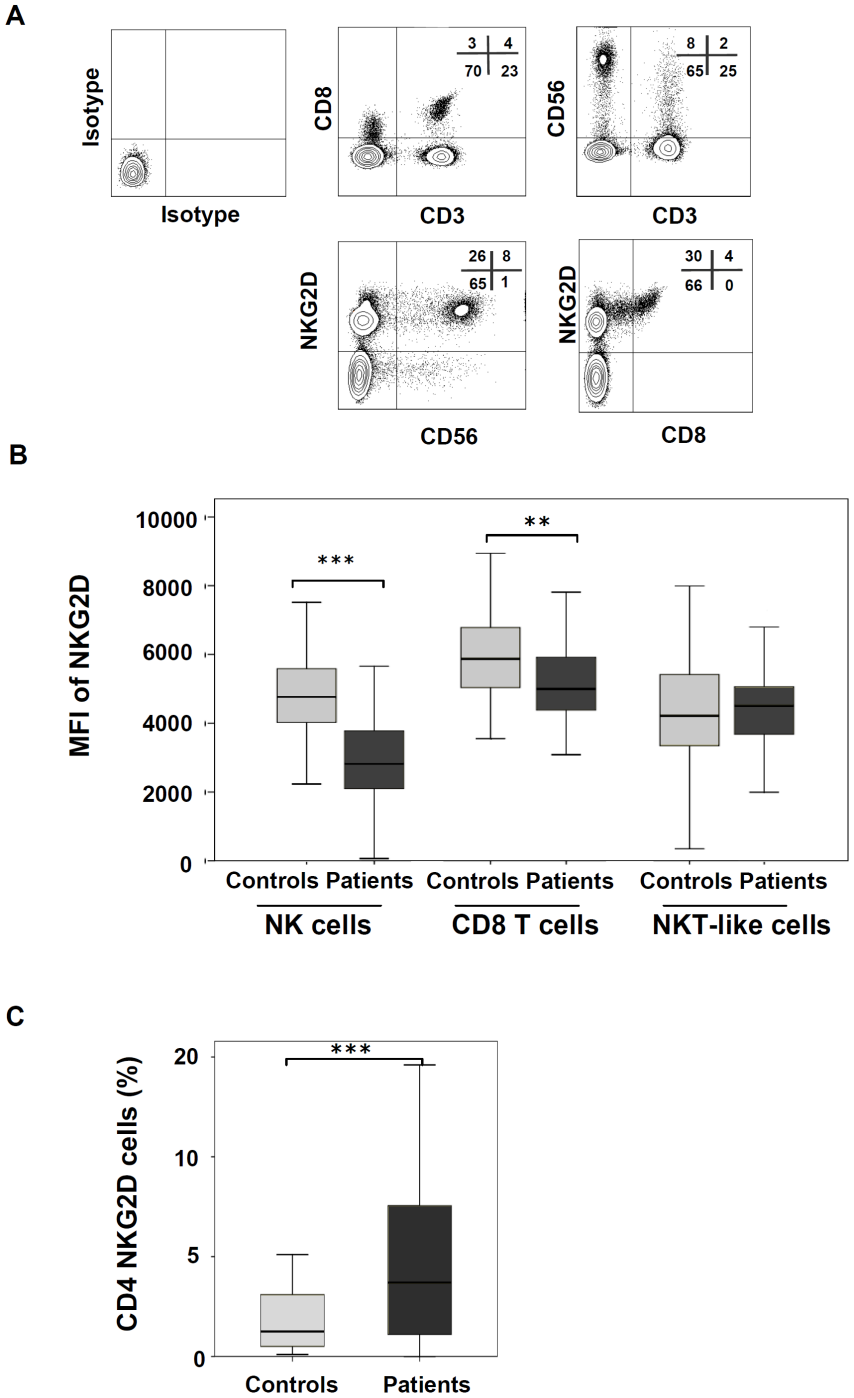
Expression of NKG2D on immune cells of CLL patients. (A) PBMCs obtained from CLL patients were stained with CD3-, CD4-, CD8-, CD56- and NKG2D-conjugated antibodies. Flow cytometry analysis of one representative patient is shown. (B) The figure shows the comparison of the MFI of NKG2D surface expression in NK cells, CD8 T cells and NKT-like cells between patients and controls. (C) Comparison of the percentage of NKG2D+CD4 T cells between patients and controls. (**p<0.01; ***p<0.001).

Flow cytometry analysis showed that NKG2D was expressed on T and NK cells, but it was absent in B cells (CD19+) of patients and controls (Fig. 4A). Compared with controls, NKG2D expression was markedly reduced on NK cells of patients (mean fluorescence intensity (MFI) of 2878 vs. 4778, p<0.0001), and on CD8 T cells (5202 vs. 5882, p = 0.003) (Fig. 4B). The percentage of CD4+NKG2D+ T cells was significantly higher in patients than in controls (median of 8% vs. 2.7%, p = 0.001) (Fig. 4C). CD4+NKG2D+ T cells of CLL patients comprised a heterogeneous population of cells, as 51% of them were CD4+CD8+, 18% were CD4+CD56+ and 30% lacked CD8 and CD56 expression.

### The expression of NKG2D on NK cells was reduced in patients with progressive and advanced disease

**Figure 5:**
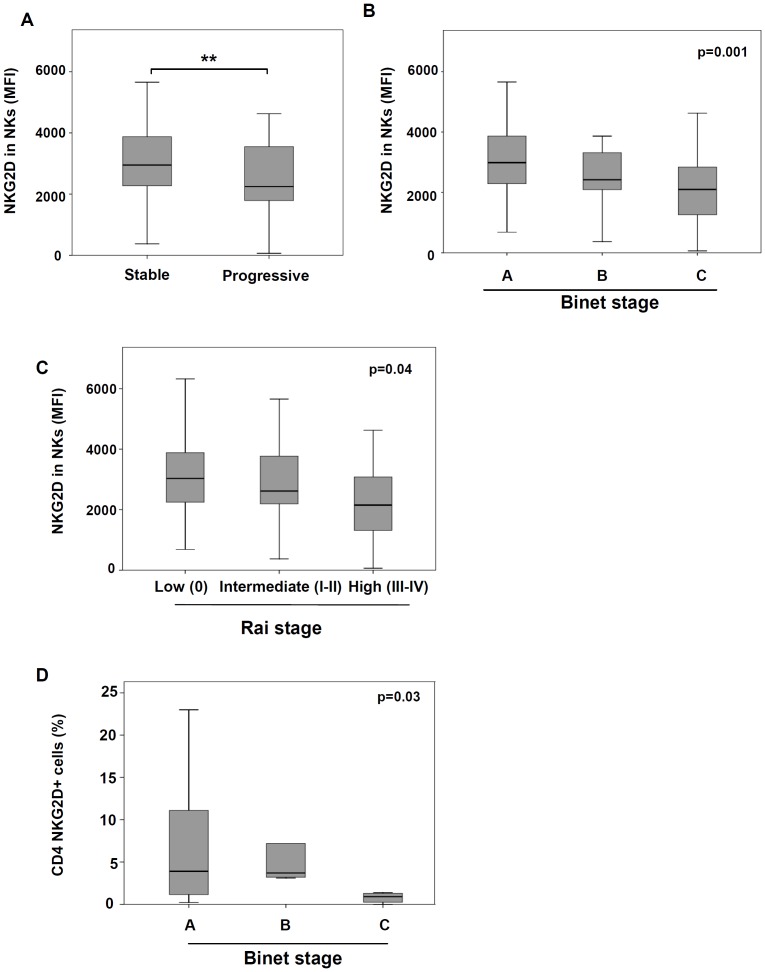
Association between clinical parameters of CLL patients and the expression of NKG2D. NKG2D expression on NK cells was analyzed in patients stratified by the existence of stable and progressive disease (A), Binet stage (B) and Rai stage (C). The percentage of NKG2D+CD4+ T cells in CLL patients was analyzed according to the Binet stage. (**p<0.01).

The association between NKG2D expression and the clinical characteristics of CLL patients was next analyzed. NKG2D expression on NK cells was significantly decreased in patients with progressive disease (p = 0.017), in patients with advanced Binet stage (p = 0.001) and in high risk patients (Rai stage III and IV) (p = 0.04) (Fig. 5A, B and C). Further, the expression of NKG2D receptor on NK cells negatively correlated with the number of affected lymph nodes (r = −0.254, p = 0.01) and ECOG (r = −0.353, p<0.001); but it directly correlated with the sera levels of lactate dehydrogenase (r = 0.277, p = 0.007). The percentage of CD4+NKG2D+ T cells was decreased in patients with advanced Binet stage disease (p = 0.03) (Fig. 5D).

### The expression of NKG2D and the cytotoxic activity of NK cells may be restored in CLL patients

**Figure 6:**
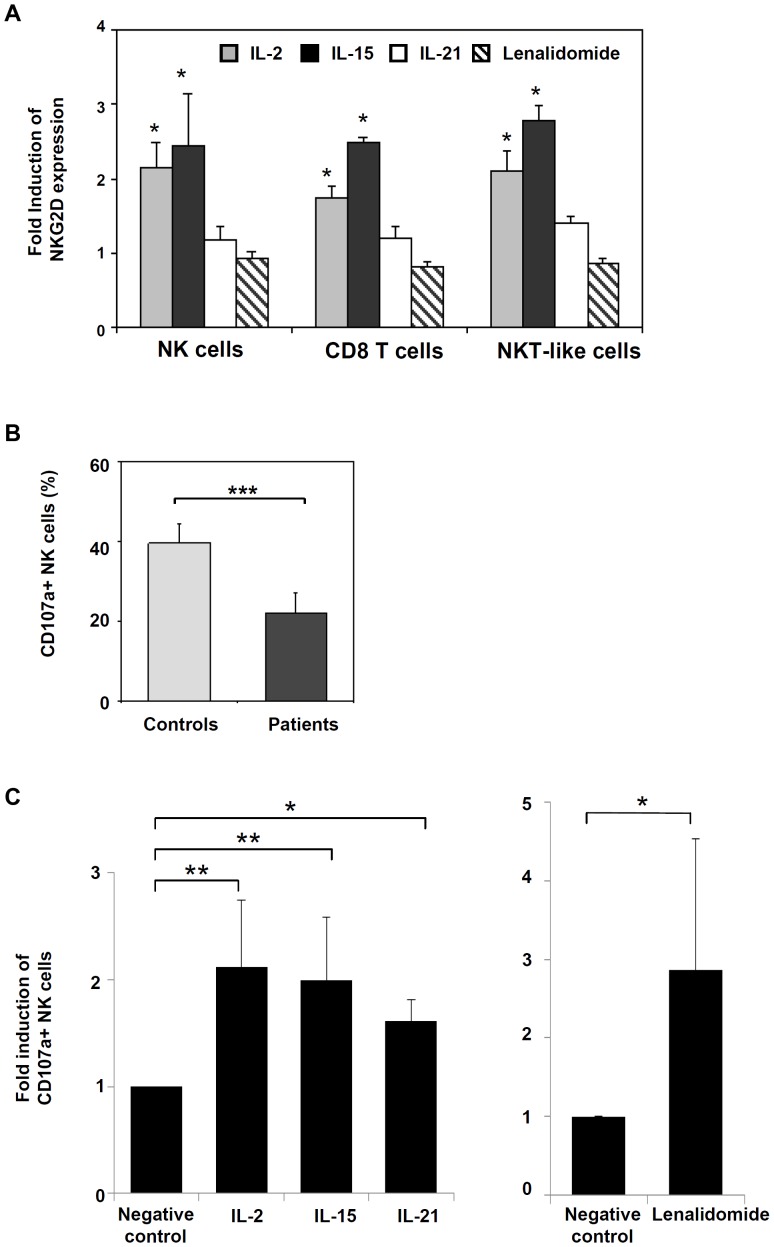
Effect of immunomodulatory molecules in the expression of NKG2D and the cytotoxic activity of NK cells of CLL patients. (A) PBMCs purified from CLL patients were incubated with IL-2 (5 ng/ml), IL-15 (25 ng/ml), IL-21 (20 ng/ml) for 48 hours or lenalidomide (1 µM) for 7 days and the expression of NKG2D on NK cells, CD8 T cells and NKT-like cells was analyzed by flow cytometry. Bars represent the mean and the standard deviation of the fold induction of NKG2D expression. (*p<0.05). (B) PBMCs from healthy donors and CLL patients were incubated with K562 cells at an E:T ratio of 5∶1 for 4 hours. The expression of CD107a in NK cells was evaluated by flow cytometry. The bars represent the mean and the standard deviation of the percentage of CD107a+ NK cells obtained in controls and patients. (C) PBMCs from CLL patients were incubated with IL-2 (5 ng/ml), IL-15 (25 ng/ml), IL-21 (20 ng/ml) for 48 hours or lenalidomide (1 µM) for 7 days, and then they were incubated with K562 cells at an E:T ratio of 5∶1 for 4 hours. The expression of CD107a in NK cells was evaluated by flow cytometry. (*p<0.05**; p<0.01; ***p<0.001).

We next studied whether NKG2D expression can be induced upon exposure to immunomodulatory molecules. Treatment of PBMCs obtained from CLL patients with IL-2 and IL-15 for 48 hours significantly up-regulated the expression of NKG2D on NK cells and CD8 T cells (Fig. 6A). No effect on NKG2D expression was observed with IL-21 or lenalidomide, even after 14 days of treatment (not shown).

Further, CD107a degranulation assay showed that NK cells of CLL patients had a diminished cytotoxic activity compared with healthy controls (Fig. 6B). Nevertheless, their cytotoxic activity was significantly enhanced when immune cells were previously stimulated with IL-2, IL-15, and IL-21 for 48 hours or lenalidomide for 7 days (Fig. 6C).

## Discussion

Here, we analyzed the immune characteristics of a cohort of previously diagnosed CLL patients. The most relevant results of our study are: (i) CD8 T cells were increased at diagnosis of CLL, and the relative number of CD8 T cells at diagnosis was associated with the clinical outcome of the patients. (ii) This initial expansion of CD8 T cells decreased overtime with the evolution of the disease, and it was not associated with the chemotherapy treatment. (iii) The number of NK cells was significantly expanded overtime in CLL. (iv) The expression of NKG2D was decreased on NK and CD8 T cells, but it was increased on CD4 T cells of previously diagnosed CLL patients. (v) The reduction of NKG2D expression on NK cells was further enhanced in patients with advanced and progressive disease. (vi) The expression of NKG2D and the cytotoxic activity of NK cells of CLL patients were restored with immunomodulatory molecules and cytokines.

The expansion of anti-tumor immune cells, mainly CD8 T cells and NK cells, has been described in several haematological malignancies [17]–[23]. Nevertheless, the expansion of T cells has not been observed in a pre-leukemic monoclonal B lymphocytosis [24], suggesting that the expansion of immune cells in CLL may be associated with the transformation of B cells. This is in agreement with the fact that we observed a significant correlation between the number of leukemic cells at diagnosis and the number of T and NK cells [24]. This expansion of CD8 T cells is associated with the clinical outcome suggesting that CD8 T cells may modify the progression of the disease. However, the potential anti-leukemic role of these expanded T cells remains to be elucidated.

Additionally, we further observed that the initial expansion of CD8 T cells decreased overtime after the diagnosis of the disease, reaching values similar to those observed in healthy controls. In the course of progression of CLL, patients develop multiple immune defects, which are associated with a severe immunodeficiency [7]. In agreement, the deterioration of the immune system observed in our study was associated with a significant increase of second malignancies in CLL patients. This may probably reflect the consequence of the immune evasion mechanisms developed by leukemia cells [25].

NK cells were also expanded in CLL patients at the diagnosis of the disease, but, unlike T cells, their number was kept elevated when they were re-evaluated after the diagnosis. In spite of the fact that the expansion of NK cells in CLL patients has been associated with better prognosis [5], we could not find any correlation between NK cell numbers and the prognosis of the patients. In agreement, NK cell activity in CLL patients is highly impaired and leukaemia cells are highly resistant to NK cell cytotoxicity [26], [27]. In this scenario, we observed a down-regulation of NKG2D expression that may contribute to the deficiency of NK cell function. This decrease of NKG2D expression was further augmented in patients with advanced disease, suggesting that leukaemia cells may be involved in the repression of NKG2D expression. In agreement, we previously reported that the reduction of NKG2D expression in CLL may be due to the chronic exposure to released soluble NKG2D ligands [15].

Additionally, we observed a profound deregulation of the expression of NKG2D on other immune cells. In this sense, NKG2D levels slightly, but significantly, decreased in CD8 T cells. Further, an increase of NKG2D expression on CD4 T cells was also detected. Low levels of NKG2D+CD4+ T cells are present in healthy individuals, but this population may be expanded in some chronic and autoimmune diseases [28]–[30]. NKG2D+CD4+ T cells with both immunosuppressive and anti-tumor activity have been described. Our results suggest that NKG2D+CD4+ T cells are a heterogeneous population of cells in CLL, but the increase of these cells in early stage Binet patients argues against an immunosuppressive role for this population.

Remarkably, our findings indicate that the impairment of NKG2D expression on immune cells of CLL patients is reversible. Thus, the expression of NKG2D in cytotoxic cells of CLL patients can be restored upon treatment with cytokines and immunomodulatory drugs [31]–[34]. IL-2 and IL-15 induced NKG2D expression on immune cells, and stimulated NK cell cytotoxicity. IL-21 and lenalidomide also stimulated the cytotoxic activity of NK cells; however, independently of the induction of NKG2D expression.

Overall, our experiments indicate that a profound deregulation of the immune system occurs with the progression of CLL. Noteworthy, the expansion of NK cells, and the reversibility of their defects, provides new opportunities for immunotherapeutic intervention in this disease.
